# Parental supervision positively impacts children’s economic prospects two decades later: A prospective longitudinal study

**DOI:** 10.1371/journal.pone.0286218

**Published:** 2023-05-24

**Authors:** Ellen W. McGinnis, Julia Halvorson-Phelan, Lilly Shanahan, Tong Guangyu, William Copeland

**Affiliations:** 1 Department of Psychiatry, University of Vermont, Burlington, Vermont, United States of America; 2 Department of Biostatistics, Yale University, New Haven, Connecticut, United States of America; 3 Psychology & Jacobs Center for Productive Youth Development, University of Zurich, Zurich, Switzerland; COMSATS University Islamabad - Lahore Campus, PAKISTAN

## Abstract

**Importance:**

Upward income mobility is associated with better health outcomes and reduced stress. However, opportunities are unequally distributed, particularly so for those in rural communities and whose family have lower educational attainment.

**Objective:**

To test the impact of parental supervision on their children’s income two decades later adjusting for parental economic and educational status.

**Design:**

This study is a longitudinal, representative cohort study. From 1993–2000, annual assessments of 1,420 children were completed until age 16, then followed up at age 35, 2018–2021, for further assessment. Models tested direct effects of parental supervision on child income, and indirect effects via child educational attainment.

**Setting:**

This study is an ongoing longitudinal population-based study of families in 11 predominately rural counties of the Southeastern U.S.

**Participants:**

About 8% of the residents and sample are African American and fewer than 1% are Hispanic. American Indians make up 4% of the population in study but were oversampled to make up 25% of the sample. 49% of the 1,420 participants are female.

**Main outcomes and measures:**

1258 children and parents were assessed for sex, race/ethnicity, household income, parent educational attainment, family structure, child behavioral problems, and parental supervision. The children were followed up at age 35 to assess their household income and educational attainment.

**Results:**

Parental educational attainment, income, and family structure were strongly associated with their children’s household income at age 35 (e.g., *r* = .392, *p* < .05). Parental supervision of the child was associated with increased household income for the child at age 35, adjusting for SES of the family of origin. Children of parents who did not engage in adequate supervision earned approximately $14,000 less/year (i.e., ~13% of the sample’s median household income) than those who did. The association of parental supervision and child income at 35 was mediated by the child’s educational attainment.

**Conclusion and relevance:**

This study suggests adequate parental supervision during early adolescence is associated with children’s economic prospects two decades later, in part by improving their educational prospects. This is particularly important in areas such as rural Southeast U.S.

## Introduction

Childrens’ opportunities in adulthood are associated with their family of origin’s socioeconomic status (SES) [1–3]. Adulthood earnings are correlated at *r* = .40 to .50 with their parents’ household earnings [4–6]. Income mobility [7] is an important metric of economic opportunity [8] and is associated with improved midlife wellbeing, and higher income [9].

Opportunities for upward income mobility are not distributed equally across SES. Children from rural communities and whose families are less educated are less likely to improve their lot [10, 11]. Those who advance economically generally have higher scores on cognitive tests and lower levels of externalizing behavior from an early age [12]. Identifying familial determinants of income mobility in children from disadvantaged families is a public health priority.

Parenting behaviors are proven modifiable factors affecting children’s economic mobility. Upward mobility is associated with increased one-on-one time and verbal interactions [8, 13–15], more age-appropriate modeling and scaffolding [16, 17] and with parents who influence their child’s decisions to align with their perception of success [8, 18], while harsh parenting is associated with worse economic outcomes [15, 19]. Parental behavior research has overwhelmingly focused on early childhood [20, 21]; and it is unknown whether parenting behavior in adolescence has similar effects.

During middle and high school, academic engagement and motivation often decline [22, 23]. Knowledge of a child’s whereabouts and daily parent-child engagement are linked to decreased adolescent delinquency [24] and school dropout [25], and greater academic engagement and achievement [26–28] and therefore, parental supervision during adolescence may influence later economic functioning [29]. For example, college completion predicts income mobility more accurately than race, ethnicity, college major, financial aid, or gender [30] and this “return on college investment” has only increased in the US in the last 50 years [18]. However, parental supervision is also associated with family SES such that job requirements, parent presence, and other demands constrain its capacity [31, 32]. This analysis uses a prospective-longitudinal study to test whether parental supervision and education contribute to improved economic prospects for children after adjusting for family SES.

## Methods

### Procedure

The Great Smoky Mountains Study is a longitudinal, representative study of children in 11 predominantly rural counties of North Caroline [1]. Three cohorts of children, ages 9, 11, and 13 at intake, were recruited from a pool of 12,000 children using a household equal probability, accelerated cohort design. where each cohort reaches a given age in a different year, controlling for cohort effects. Potential participants were randomly selected from using a household equal probability design. Participants were the screened for risk of psychopathology; participants screening high were oversampled in addition to a random sample of the rest. About 8% of area residents and sample are African American and fewer than 1% are Hispanic. American Indians make up 4% of the study but were oversampled to constitute 25% of the sample. This resulted in N = 1,420 participants (49% female). Sampling weights are applied to adjust for differential probability of selection. The statistical estimates presented are representative of the population from which the sample was drawn.

Annual assessments were completed until the children reached age 16 (6,674 observations of 1,420 participants; 1993 to 2000) and again in their late 30s (interquartile range: 35–38, 1013 participants; 2018–2021). Interviews were completed by a parent figure and the participant to age 16, and by the participant thereafter. Before interviews, the parent and child signed informed consent/assent forms. The study protocol and consent forms were approved by the Duke University Medical Center Institutional Review Board. Each respondent received an honorarium for participating. The current analysis only includes data from early adolescence (ages 13–16).

## Measures

### Items assessed in childhood (13–16 years)

All items were assessed using the Child and Adolescent Psychiatric Assessment (CAPA) interview [33, 34], administered to each participant’s primary caregiver annually from study entry until 16. Items have been shown to display test-retest reliability and discriminant validity [33–35].

#### Child demographics

Child race/ethnicity and sex were parent-reported.

#### Family structure

The parent reported whether they were single (0 ‘not single,’ 1 ‘single’). If single when the child was 13–16, they were coded as “single,” and as “not single” otherwise.

#### Parental household income

Household income between child ages 13 and 16 was reported by a parent each year between 1993–2000 on the following scale: 0 = No Income; 1 = 0,001–5,000; 2 = 5,001–10,000; 3 = 10,001–15,000; 4 = 15,001–20,000; 5 = 20,001–25,000; 6 = 25,001–30,000; 7 = 30,001–35,000; 8 = 35,001–40,000; 9 = 40,001–45,000; 10 = 45,000–50,000; 11 = 50,001–55,000; 12 = 55,001–60,000; 13 = 60,001 or more. The maximum parental household income reported was used and adjusted for 2021 inflation in descriptive conclusions to better make comparisons with child income assessed around 2021 (*Consumer Price Index Data from 1913 to 2022 | US Inflation Calculator*, 2008).

#### Parental educational attainment

The maximum attainment by any parent in the home was used from the following scale: 1 = 0–8 years; 2 = Some high school; 3 = GED or high school equivalency; 4 = High school degree; 5 = Post high-school training (vocational, technical, job training); 6 = Some college (0–2 years); 7 = 2 year associate degree; 8 = Some college (2–4 years); 9 = four year college degree; 10 = Some graduate or professional school training; and 11 = Completed graduate or professional degree.

#### Inadequate parental supervision

Inadequate parental supervision is defined in the CAPA as “parent fails to provide sufficient supervision as shown by frequent lack of knowledge of child’s whereabouts, activities, or company; and/or fails to maintain effective control/ or disciplinary strategies; and/or is not concerned, or does not attempt to intervene, when child’s behavior is deviant, or likely to lead him/her into trouble.” Parents were administered prompts including, “Do you expect X to let you know where s/he is?”, Do you always know where X is when s/he isn’t home?” and “What happens when X doesn’t want to do what you say?”. Interview coded responses into 3 categories of 0 ***= “***Appropriate supervision/control for age and circumstances,” 1 = “Whereabouts of child not known at least once per week; or parent unable to exercise effective control at least once per week,” or 2 = “Whereabouts of child unknown at least 5 times per week; or parent usually (>50% of the time) unable to exercise effective control.” The maximum score across caregivers at child ages 13 to 16 was used for subsequent analyses (Range: 0–2). Extreme groups, 0 and 2, will be referred to as “adequate” and “inadequate” supervision, respectively.

#### Behavioral symptoms

Inadequate supervision included the possibility of endorsement due to parent’s inability to “maintain effective control over child.” Child behavioral symptoms were included as a covariate as the focus of this analysis is on parent’s–not child’s—behavior. Behavioral symptoms included the nine symptoms of Oppositional Defiant disorder (ODD) and fifteen symptoms of Conduct Disorder (CD), then assessed using the CAPA (parent-report). If a symptom was endorsed by either parent or participant, it was counted. All symptoms summed to create a behavioral symptom total at age 16.

### Adult outcomes (35 years)

#### Income

Household income was reported at age 35 in 2018–2021 on the scale: 1 ’$0–25,000’; 2 ’$25,001–50,000’; 3 ‘$50,001–75,000’; 4 ’$75,001–100,000’; 5 ’100,001–150,000’; 6 ‘$150,001+’.

### Potential mediator (35 years)

#### Participant education

Education was reported at age 35 on the following scale: 0 ’did not finish high school;’ 1 GED or high school equivalency;’ 2 ’Associates Degree;’ 3 ’bachelor’s degree;’ and 4 ’Graduate program’.

*Cognition*. A subsample of participants included in these analyses (n = 510) were administered the Wechsler Abbreviated Scale of Intelligence-Second Edition at age 30 (Wechsler, 2011). Given the role of intelligence in life outcomes [36], we were able to test full IQ as a covariate using this subsample.

### Missing data

Eighty-two percent (ranging from 74–94%) of all possible interviews were completed across waves. All participants completed at least 1 assessment by age 16 (period parental supervision assessed); 94.3% had ≥3 assessments (median = 7). 76% of eligible participants had adult assessments at age 35 (when outcomes were assessed). There were no differences in attrition from age 16 to 35 by inadequate parent supervision *t*(452.3) = -.825, *p* = .410, or childhood behavioral symptoms *t*(1278) = -.678, *p* = .498. those who dropped out had parents with lower education *t*(1256) = 5.24, *p* < .001, and lower household income *t*(501.9) = -5.48, *p* < .000 at age 16, common in longitudinal research [37, 38]. The analyses were completed using multiple imputations with 10 datasets under the missing-at-random assumption. Results were synthesized using Rubin’s rule [39, 40].

### Statistical models

Each participant was assigned a sampling weight inversely proportional to their probability of selection. all percentages provided are weighted and sample sizes are unweighted in descriptive tables. We incorporate the sampling weight into a series of multivariate ordinal logistic regression models to establish the relationship between early adolescent characteristics and income levels at age 35. Models adjusted for familial/demographic covariates including child sex, race/ethnicity (white, African American, American Indian), parent structure, parent education, and household income. In subsequent models, we included parenting-relevant variables: inadequate supervision and child behavioral symptoms. Mediation tests (Sobel’s test, Aroian’s test, and Goodman’s test) were performed to evaluate indirect effects of parenting variables on child income during adulthood [41]. The confidence intervals of the effect estimates were derived from 5,000 bootstrap samples, and the proportional odds assumptions for the ordinal logistic regression models were tested and found violated for the primary analytical models. Thus, an additional simulation-based linear regression analysis was performed based on 1000 randomly generated datasets with income values from a mixture of interval-specific uniform distributions (< = $150k) and a truncated normal distribution (>$150k with SD = $10k) for the income variable. Changing dispersion of the standard deviation parameter yields similar findings. For this analysis, 95% confidence intervals were obtained from the 2.5%- and 97.5%-point estimates based on the 1000 datasets. This approach provided dollar amount estimates to the direct and indirect effect of parenting variables. All analyses were performed using R 4.0.1. Ordinal logistic regression models were fitted using “MASS” package; multiple imputation and result synthesis were performed with the “mice” package.

## Results

### Descriptive analyses

Descriptive analyses provided in **Table 1**. The sample is ~50% female, majority white. 25% of parents were single, ~30% had bachelor’s degrees, and average household income adjusted for inflation was ~$56,491 in 2021 (when participant adult income was assessed). Most parents knew where their children was at all times, and the average number of child behavior symptoms was low. ~38% of participants attained at least a bachelor’s degree, and their average household income in 2021 was $60,250. The correlation between parental and participant adult income was *r* = .37, *p* < .05.

**Table 1:** Frequencies and means of family demographics, parenting behaviors, participant education and participant adult income.

		*Weighted % or M*	Unweighted N or SD
**Family Demographics Model**			
Participant Sex	Female	48.9%	584
	Male	51.1%	713
Participant Race	White	89.8%	884
	African American	6.2%	74
	American Indian	4.0%	339
Family Structure	Not Single	74.8%	899
	Single	25.2%	357
Parental Education	Highschool or less	25.6%	582
	Some College	37.6%	360
	4 Year College Degree	17.5%	165
	Graduate Degree	19.4%	144
Parental Income	Average (2021 inflation)	$56,491	$15,750
**Parenting Behavior Model**			
Participant Behavior Symptoms	Sum of ODD and CD behaviors at age 16.	0.61	1.23
Parental Supervision (Max)	Adequate Supervision	77.2%	893
	Whereabouts unknown at	9.8%	134
	least once per week		
	Inadequate Supervision	13%	229
**Mediation Model**			
Participant Education	High school or less	43.9%	564
	Some College	17.6%	191
	4 Year College Degree	25.9%	173
	Graduate Degree	12.5%	85
**Outcome at 35 Years**			
Participant Adult Income	Avg. in year 2021	$60,250	
	$0-$25,000	17.1%	246
	$25,001-$50,000	15.9%	214
	$50,001-$75,000	19.2%	164
	$75,001-$100,000	15.4%	137
	$100,001-$150,000	21.5%	128
	>$150,000	10.8%	84

Note: ODD is Oppositional Defiant Disorder; CD is Conduct Disorder

#### Simple family demographics model

We tested a model with all family demographic variables predicting adult household income (**Table 2**). Neither participant gender nor race (African American or American-Indian compared to white) reached statistical significance. Parental education and income were significantly associated with participant adult income. For every ~2 years of parental post-high school education, there was a yearly increase of $4,464 (CI: $4,206-$4,729) in the child’s adult income. For every $5,000 increase in parental income, there was a yearly increase of $4,199 (CI: $4,011-$4,388) in the child’s adult income. Being raised in a single parent household appeared to have a positive effect on income mobility in the multivariate regression model, however, post hoc analyses revealed family structure and parental income were confounded; altering the direct effect of family structure to represent only 3% of single parents in the sample who earned more than the mean household earnings ($56,491) and is not discussed hereafter.

**Table 2:** Multivariate ordinal logistic regression model of family demographics predicting to participant adult income.

	Odds Ratio	95% Confidence Intervals		*P Value*
		*LL*	*UL*	
Participant Gender				
Male vs. Female	1.10	.84	1.43	0.505
Participant Race				
African American vs. White	0.53	.28	1.00	0.050
American Indian vs. White	0.60	.35	1.03	0.064
Family Structure				
Single	1.54	1.13	2.11	0.007
Parental Education	1.15	1.07	1.23	0.000
Parental Income	1.15	1.10	1.20	0.000

#### Test of parental supervision

We added parental supervision and child behavioral symptoms to the family demographic model (**Table 3**). Participant behavioral symptoms were included to account for inadequate supervision due to challenging child behavior. Parental supervision during early adolescence was significantly associated with adult income. For every decrement in adequate parental supervision (0 to 1, or 1 to 2), there was a decrease of $7,087 (CI: -$7,823- -$6,274) in the child’s adulthood income. Participant behavior symptoms were also associated with adult income: For each behavioral symptom during adolescence, adult income decreased by $4,114 (CI: -$4,638- -$3,562).

**Table 3:** Multivariate ordinal logistic regression model of family demographics and parenting variables predicting to participant adult income.

	Odds Ratio	95% Confidence Intervals		*P Value*
		*LL*	*UL*	
Participant Gender				
Male vs. Female	1.19	0.81	1.76	0.374
Participant Race				
African American vs. White	0.52	0.24	1.11	0.091
American Indian vs. White	0.60	0.43	0.85	0.004
Family Structure				
Single vs 2 Parent Household	1.67	1.04	2.68	0.035
Parental Education	1.15	1.05	1.26	0.002
Parental Income	1.14	1.07	1.21	0.000
Parental Supervision	0.77	0.59	1.00	0.046
Participant Behavior Symptoms	0.80	0.66	0.96	0.015

To account for the potential contribution of participant intelligence related to inadequate supervision, and/or differences in resulting adult income full IQ was included in analyses for a subsample of participants (n = 510) for whom were administered IQ testing during the study. In this subsample controlling for all previously stated factors, full IQ was significantly associated with adult income (B = .06, SE = .01, p < .001), however, inadequate parental supervision continued to significantly to predict adult income (B = -.342, SE = .17, p < .041).

#### Test of mediation by educational attainment

**Fig 1:**
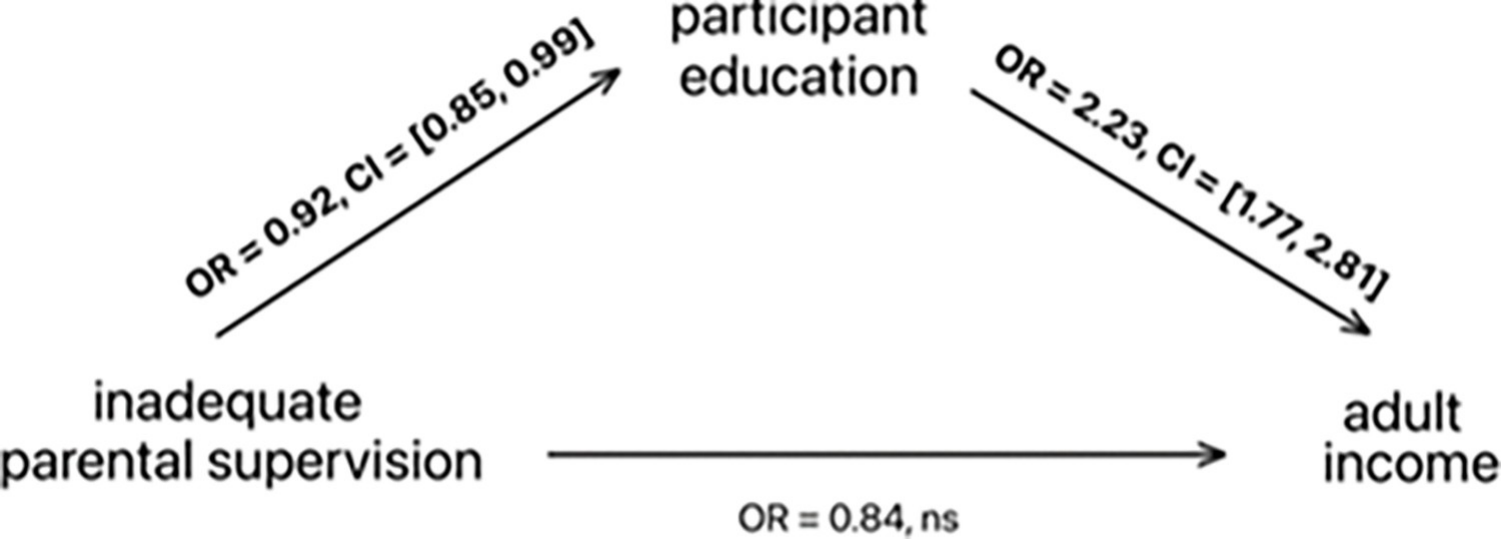
Mediation pathway demonstrating the impact of inadequate parental supervision (at ages 13–16) adolescence on participant adult income (at age 35) via participant educational attainment (at age 35).

We tested whether the association between parental supervision and participant adult income was mediated by participant education. Adjusting for all covariates from previous models, parental supervision was strongly associated with participant adult education (Odds Ratio = .69, Bootstrap CI: .57-.84, *p* = .0001). Participant education was strongly associated with participant adult income. The inclusion of participant education in the model rendered the direct effect of parental supervision non-significant (Odds Ratio = .84, Bootstrap CI: .62–1.12, *p* = .237, see **Table 4**. Mediator tests [41] all suggest education is a statistically significant mediator (Sobel’s test, p = .044, Aroian test, p = .046, Goodman test, p = .042). See **Fig 1**. for mediation pathway.

**Table 4:** Multivariate ordinal logistic regression model of family demographics, parenting variables, and participant education predicting to participant adult income.

	Odds Ratio	95% Confidence Intervals		*P Value*
		*LL*	*UL*	
Participant Gender				
Male vs. Female	1.45	0.95	2.81	0.082
Participant Race				
African American vs. White	0.58	0.28	1.20	0.141
American Indian vs. White	0.67	0.48	0.95	0.024
Family Structure				
Single vs 2 Parent Household	1.30	0.79	2.12	0.017
Parental Education	1.06	0.96	1.17	0.227
Parental Income	1.08	1.01	1.16	0.017
Parental Supervision	0.84	0.63	1.12	0.237
Participant Behavior Symptoms	0.84	0.70	1.00	0.047
Participant Adult Education	2.23	1.77	2.81	0.000

## Discussion

These analyses examine the effect of parent supervision during adolescence on income mobility two decades later, in a rural region with little economic mobility [42]. Our analyses suggest parental supervision is significantly more important than family status for adult income. Having adequate parental supervision during adolescence in the late 1990s resulted in a lifetime income difference of ~$219,870 (CIs: $172,290 to $261,180) between ages 35–65 (without pay increases). This lifetime income difference is equivalent to ~1–2 extra years of parent education, or an additional $10,000 in annual parental household income. Parental supervision has been associated with positive academic and behavioral outcomes and child well-being [25–28], albeit additional potentially confounding factors still must be explored (i.e., morbidity, access to healthcare, personality). Bearing these in mind, our results may suggest positive cascade effects of parental supervision beyond adolescence and on income two decades later, subsequently influencing the child’s social mobility.

The association between parental supervision during early adolescence and adult income is mediated by education. This is supported by previous research on mechanisms where parental supervision may be beneficial. Studies show more parental supervision is associated with more investment in homework assignments, class schedules, extracurricular activities, and academic pursuits [43]. More parental supervision may protect against truancy and delinquency [24] and staying academically engaged can help students achieve higher education [26–28]. In the U.S., substantial economic research has shown a high return of post-high school education, including nearly 84% greater average earnings for college graduates as compared to nongrads [44]. Thus, there are benefits to examining how parental behavior can help children maximize educational attainment. Academic engagement naturally decreases for most students during early adolescence [45], making this period particularly sensitive and an important window for parental supervision.

Children from rural communities whose parents are less educated are less likely to achieve upward mobility than those in metropolitan areas whose parents are more educated [10, 11]. Rural Appalachia represents counties with some of the lowest rates of upward mobility in the country, where of the bottom fifth income percentile, only 5% reach the top fifth income percentile (compared to up to 16.8% in other regions). Determinants include high degrees of racial segregation, high rates of teenage pregnancy, poor public schools, and low rates of college attendance [11]. The positive effect of parental supervision is noteworthy as it may be more modifiable than other determinants. Despite known associations between family financial resources and parenting behaviors [31, 32], parental supervision had a weak correlation with parental income (r = -.13, p < .01) or parental education (r = -.07, p < .05) in our sample. These findings can be used by parents across the socioeconomic spectrum to support child outcomes.

### Limitations

The prospective, intergenerational study design minimizes the risk of reporting bias that affects retrospective studies [46]. Simultaneously, there was differential attrition related to lower household income and parental educational attainment. This was addressed by multiple imputed datasets to include observations with missing data. This study relied upon self-report for household income, as misclassification bias might have occurred, and which may exhibit improved accuracy with multiple informants or public records. The primary measure—parental supervision—was derived from a single item. This was coded based on a series of primary and secondary probes until the interviewer was certain they could code the response appropriately. This item was coded based on parental knowledge of child whereabouts and ability to mediate child behavior. We adjusted for childhood behavior to minimize the likelihood that results would be due to unmanageable child behavior. There are several potentially confounding factors that we did not account for which likely contribute to income mobility which should be examined in future works including morbidity, access to healthcare, community violence, personality factors, and genetics related to parental supervision and/or income mobility. Additional detail about parental supervision would have allowed us to explore curve-linear relationships between parental supervision and child outcomes as seen elsewhere [47]. This study was not representative of the United States, but of individuals in the Greater Smoky Mountain region, where specific groups (i.e., American Indians) were over-represented and therefore may be represent other communities.

## Conclusion

A key part of the American dream is children’s potential to rise above their circumstances. This study suggests parents across the socioeconomic spectrum, parental supervision of their teenagers is an important factor in their future economic welfare. This had an equivalent effect on offspring’s adult lifetime earnings as (hypothetically) raising their own income by $10,000 per year. However, examination of more potentially confounding variables (such as cognitive ability and personality factors) must occur prior to concluding that simple improvement of parental supervision itself would increase future adult earnings. We know financial resources improve child outcomes, and this study suggests parental behavior likely also has a role. Based on our findings, policy could be implemented to focus on helping parents in their capacity to supervise their children via providing more family and community resources, and/or psychoeducation. For instance, a brief video intervention increased awareness of adolescent risk behaviors and led to increases in supervision and parent-adolescent concordance in one study [48].
