# Correction: Parental supervision positively impacts children’s economic prospects two decades later: A prospective longitudinal study

**DOI:** 10.1371/journal.pone.0304892

**Published:** 2024-05-31

**Authors:** Ellen W. McGinnis, Julia Halvorson-Phelan, Lilly Shanahan, Guangyu Tong, William Copeland

The fourth author’s name is incorrectly switched. The correct name is: Guangyu Tong. The correct citation is: McGinnis EW, Halvorson-Phelan J, Shanahan L, Tong G, Copeland W (2023) Parental supervision positively impacts children’s economic prospects two decades later: A prospective longitudinal study. PLoS ONE 18(5): e0286218. https://doi.org/10.1371/journal.pone.0286218.

## References

[1] McGinnis EW, Halvorson-Phelan J, Shanahan L, Guangyu T, Copeland W (2023) Parental supervision positively impacts children’s economic prospects two decades later: A prospective longitudinal study. PLoS ONE 18(5): e0286218. 10.1371/journal.pone.0286218 37224161 PMC10208455

